# The Military (Alexander) Hospital. Warsaw

**Published:** 1892-09-03

**Authors:** 


### CONSTRUCTION AND MANAGEMENT.

### THE MILITARY (ALEXANDER) HOSPITAL, WARSAW.

Following the main thoroughfare of the town, with its tramway line, with its well-paved streets and handsome buildings, the roadway opens out to the left upon a park, with booths for the sale of kwas, oranges, and cakes. A little further away, cut off from the public by a lofty wooden paling, is a long, regular building, which to the practised eye must be either a barrack or a hospital. And, indeed, the word Gospitalja standing above the doorway, and the red cross on the wall to the side, marks the character of the institution.

The circuit of the enclosure, partly a railing, partly a wooden paling, must be at least half or three-quarters of a mile. On the side, remote from the town, the estate sinks precipitously down to the banks of the Visla or Vistula, flowing from the well-wooded country to the south.

The site is rectangular, and the public entrance is in one of the shorter sides. Entering the open wooden gate without hindrance, the road divides the estate into a smaller part towards the town, on which a series of new wooden barracks are in process of erection, and a larger part overlooking the river, on which are a series of subsidiary buildings; and then the long frontage of the hospital proper, with the gateway in a central piece of two storeys. This main exposure passes back at right angles at either extremity into two wings, having a courtyard in the centre partly laid out as ornamental grounds, partly covered by the kitchen and washhouse buildings, summer barracks, and dispensary. The courtyard, it will be understood, is open towards the river.

Two rough Polish porters, engaged in cleaning out the drains, answered every request for admission with the ominous words, "Nyet! Nyet!" Patience was, however, rewarded, for Dr. Stephanovitch, head of the surgical department, accompanied by one of his juniors, both in semi-military uniform, was just returning to the town after his morning visit. He was most courteous and accessible, and gave a kind invitation to accompany him on his round next morning at 10 a.m.

A Corridor Hospital, with the Wards to one Side, on the other being Windows Overlooking the Courtyard.

The younger surgeon received his visitor next morning, conducted him through the gate to an arched space, which opened at once upon the ornamental ground. To the right and the left were the entrances to the medical and surgical sides. Mounting a few steps, one entered the ground corridor, to the left, of the surgical department. A succession of spacious wards lay on one hand, while to the other were the windows in the wall that separated from the courtyard. One quarter, which might have been a ward, was used by the medical officers as a robing room.

A little further, mounting an iron staircase, the upper floor was reached, there being in all two floors, ground floor and first story. My conductor then left me on a seat in the corridor under the window to await Dr. Stephanovitch's arrival. The corridor is very substantial, wide, and lofty. The floor is of large boards, painted chocolate and polished, and this flooring is met with throughout, except in the operation rooms and the lavatories, where it is replaced with cement concrete, covered or not with paint.

While waiting there was opportunity to observe a lady seated near with a child, whom she had brought to consult the surgeon; the hospital Sisters in black staff gowns, with white aprons and head-dress, and the cross in red upon the breast; and the patients who were convalescent, some of whom, of ancient Tartar extraction probably, looked like Japanese or Chinese. Not unfrequently there were individuals marked with small-pox. Vaccination is now obligatory in infancy, and soldiers must be re-vaccinated in Russia. The law, however, has not been in force for more than twelve or fifteen years.

Thick calico matting is laid down the centre of the corridor, and there are seats provided. The walls are painted light blue or green to the height of six feet, above that being wash; but there is no ornamentation.

A curious water reservoir, supplied by a pipe, is fixed on the wall. Three depending pipes hang over a metal trough. The pipes have no taps, but when a patient wants to fill his can, he pushes a plug from below back into the pipe, the water flows, he removes his finger, the plug falls down again by its own weight, and the pipe is shut.

The Wards.

Opposite were three wards, similar to each other, separate from each other, and opening only from the corridor. A metal plate in the doorway states that the ward contains 13,932 cubic feet, and there being 16 beds, a cubic space of about 1,000 feet is allowed per bed. The beds are arranged eight along the walls on each side. Opposite the door there are two windows and no beds. The bedsteads are of an iron framework, fitted with wooden pieces at head and foot. The usual tables and utensils stand at the bedside. Mattresses are of straw.

Heating throughout is by hot water; but besides there are two lofty stoves, rising nearly as high as the roof, covered with tiles, in the two corners towards the door.

Ventilation is effected by iron gratings, the gratings near the roof admitting fresh air, that near the floor abstracting foul air by means of a *cheminée d'appel*. This *cheminée d'appel* is well seen in the corridors. On opening the grating one finds a small fire-place with a gas-jet burning, or provided with a small fire of wood or useless bandages and the like. The chimney rises through the wall to the roof, and of course the draught established sucks foul air out of the corridor.

The walls were in oil-colour, and wash just as the walls of the corridors, and quite without ornamentation as a rule, unless for some religious symbol placed upon a bracket high up in one corner.

### Total Accommodation.

On April 28th last there were 1,300 patients in the hospital, 350 surgical, and nearly 1,000 medical. All varieties of disease are admitted; 33 per cent., however, Dr. Stephanovitch states, being tubercular. Annually, there are 200 cases of typhoid fever, and perhaps 30 cases of typhus fever, the latter being at once placed in an isolation department.

The medical service embraces 30 medical officers of the Army staff.

It was observable that the provision for typhoid cases, acute and convalescent, included a double ward; in that the two wards opened from one another, as well as opened from the corridor.

There was no opening of doors, for the wards were placed in communication with the corridor by an open door space.

The privies for this quarter are separate from those provided for general use, and, unlike these latter, are single, and arranged for immediate disinfection.

### Operation Rooms.

These include principal operating theatre, an operating-room, and a bandaging-room. The floors are of painted concrete, sinking towards a grating in the centre, and can be readily cleansed. Stores are provided for burning soiled bandages. The tables are of wood, painted in permanent colour, so that they can be readily washed. That in the large operating theatre is in three separate pieces, so that the table can be arranged and re-arranged, placed lengthwise or crosswise, where, for example, it is convenient to have the patient's arms extended; or again, as one piece does not rise so high as the others, with provision for cases where it is convenient to have one member unsupported, so that the surgeon can proceed from below as well as from above.

The instrument-table is of zinc, the instrumentarium against the wall entirely of glass and iron.

### Baths and Privies.

The water for the baths is heated by what are known in this country as "geysers." A permanent bath is provided, with a canvas bed, which can be lowered into the water.

The privies for the convalescent inmates are arranged four on one pipe, and are all flushed together at intervals, as convenient, by an attendant, in whose possession the key remains.

Smoking is permitted only in this quarter, where accordingly a few patients will mostly be found congregated.

At this point the Intendent, or House Governor, joined Dr. Stephanovitch, and hospitably offered a cigarette. He invited to come and inspect the kitchen.

### Management.

While Dr. Stephanovitch was Surgical Director, head of the surgical side, and colleague of the Medical Director, the head of the medical side, the Intendent, a lieutenant-colonel of the Russian army, was manager in all domestic concerns.

The chief of all, holding the military rank of general, met us in the courtyard, and I could not but make a profound bow when I had the honour of being introduced to that exalted personage.

### The Kitchens.

This building includes two divisions, in one of which the superior diet for officers is prepared, in the other the ordinary diet of the rank and file.

Cooking is by steam in large copper pots after the ordinary fashion.

The articles ready, for it was the dinner hour, were sampled. Excellent white bread, vegetable broth truly appetising, and a large risolle, articles from the superior *menu*, left nothing to be desired. On the other side was coarser white and brown bread, different varieties of soup, or rather broth with vegetables, and a coarser dish of cooked barley or rye, with, finally, a glass of kwas.

As I left the kitchen I could not but feel that these were among the most courteous gentlemen who ever showed me round a hospital.

### The Summer Barrack.

It is customary in Russia where the summers are very warm to transfer patients during the hot months to light wooden barracks where the atmosphere is cool. Such a barrack is located in the grounds.

Such a barrack the Intendent now entered. The approach was in the centre, and passing through the usual offices, one was in a large quarter for fifty beds. The floor was of asphalte, the walls and roofing of wood. Some patients lay, others were up and about. Wooden pillars at regular intervals supported the roof. Tables by the bedside carried medicine, milk, rye or wheaten bread, or other necessaries.

The sufferers were mostly afflicted with tubercular complaints. There was, however, a case of lepra in a small single room at one gable. The tubercles were very numerous and large in size. He was said, however, to be improving under treatment.

The visit was over, but the Intendent offering another cigarette, stepped out upon the plateau, with a view of the Vistula and the wide plain beyond. He raised his hand towards it, and the interpreter who accompanied, for the Governor only spoke Russian himself, raised his hand too, and said to me in explanation, "All that! all of it belongs to the Czar."

